# Genetic and Epigenetic Alterations of the *NF2* Gene in Sporadic Vestibular Schwannomas

**DOI:** 10.1371/journal.pone.0030418

**Published:** 2012-01-25

**Authors:** Jong Dae Lee, Tae Jun Kwon, Un-Kyung Kim, Won-Sang Lee

**Affiliations:** 1 Department of Otorhinolaryngology-Head and Neck Surgery, Soonchunhyang University College of Medicine, Bucheon, Korea; 2 Department of Biology, College of Natural Sciences, Kyungpook National University, Daegu, Korea; 3 Department of Otorhinolaryngology, Yonsei University College of Medicine, Seoul, Korea; Virginia Commonwealth University, United States of America

## Abstract

**Background:**

Mutations in the neurofibromatosis type 2 (*NF2*) tumor-suppressor gene have been identified in not only NF2-related tumors but also sporadic vestibular schwannomas (VS). This study investigated the genetic and epigenetic alterations in tumors and blood from 30 Korean patients with sporadic VS and correlated these alterations with tumor behavior.

**Methodology/Principal Findings:**

*NF2* gene mutations were detected using PCR and direct DNA sequencing and three highly polymorphic microsatellite DNA markers were used to assess the loss of heterozygosity (LOH) from chromosome 22. Aberrant hypermethylation of the CpG island of the *NF2* gene was also analyzed. The tumor size, the clinical growth index, and the proliferative activity assessed using the Ki-67 labeling index were evaluated. We found 18 mutations in 16 cases of 30 schwannomas (53%). The mutations included eight frameshift mutations, seven nonsense mutations, one in-frame deletion, one splicing donor site, and one missense mutation. Nine patients (30%) showed allelic loss. No patient had aberrant hypermethylation of the *NF2* gene and correlation between *NF2* genetic alterations and tumor behavior was not observed in this study.

**Conclusions/Significance:**

The molecular genetic changes in sporadic VS identified here included mutations and allelic loss, but no aberrant hypermethylation of the *NF2* gene was detected. In addition, no clear genotype/phenotype correlation was identified. Therefore, it is likely that other factors contribute to tumor formation and growth.

## Introduction

Vestibular schwannomas (VS) are benign tumors of the neural sheath that originate on the vestibular nerves. They occur as either sporadic unilateral tumors or bilateral tumors that are a manifestation of neurofibromatosis type 2 (NF2). The loss of both wild-type copies of the *NF2* tumor suppressor gene is common in the pathogenesis of both sporadic and NF2-related schwannomas [1], [2]. Although mutations in the *NF2* gene have been reported in 53∼70% of sporadic VS (Neff et al., 2006), genetic alterations alone cannot explain the gene inactivation.

Recently, the importance of DNA methylation in tumors has been acknowledged, and the hypermethylation of promoter-associated CpG islands is emerging as the primary mechanism of epigenetic inactivation of tumor-suppressor genes in cancer development [3], [4]. Therefore, hypermethylation in the regulatory region of the *NF2* gene has been suggested as a possible mechanism of gene inactivation [5].

Attempts have been made to correlate the clinical expression of the sporadic VS tumors with specific *NF2* mutations [6], [7], [8]. Given the heterogeneity of the clinical response to various types of mutations, no clear genotype/phenotype correlation has been established [9]. However, a recent study reported a significant relationship between tumor behavior and genetic alteration in Chinese patients [6].

The present study analyzed the genetic alterations in sporadic VS, including mutations, loss of heterozygosity (LOH), and epigenetic alterations of the *NF2* gene, and correlated these alterations with tumor behavior in Korean patients.

## Materials and Methods

### Patients

A total of 30 unrelated subjects with sporadic unilateral VS were recruited from the Department of Otorhinolaryngology - Head and Neck Surgery, Soonchunhyang University Bucheon Hospital and the Department of Otorhinolaryngology, Yonsei University Health System Hospital, Korea. The subjects included 10 males and 20 females ranging in age from 11 to 73 years (mean age, 48.3 years). Blood was collected from all patients, and tumor tissue was obtained at the time of surgery and stored at −80°C before DNA isolation. DNA was isolated from both peripheral blood leukocytes and tumor tissues. Peripheral blood leukocyte DNA was used to investigate *NF2* germ-line mutations and tumor tissue DNA was examined for somatic mutations of *NF2*. Written informed consent was obtained from all patients, and the study was approved by the ethics committee of Soonchunhyang University Hospital.

### Tumor behavior

Tumor behavior was assessed using tumor size (maximum diameter in centimeters), a clinical growth index, and proliferative activity according to the Ki-67 labeling index (LI). The clinical growth index was calculated by dividing the tumor size (maximum diameter) by the duration of the initial presenting symptom in years. To determine the Ki-67 LI, immunohistochemistry was performed on formalin-fixed paraffin sections of the VS using an anti-MIB-1 antibody followed by microscopy (Olympus, Tokyo, Japan); all nuclear-stained tumor cells were counted at 400× magnification. The mean LI was determined as the number of positively stained nuclei in three randomly chosen fields divided by the total number of cells in the selected areas. The results are expressed as the percentage of the total cell number.

### Mutational analysis

For the 30 subjects, genomic DNA was isolated from peripheral blood using a FlexiGene DNA extraction kit (QIAGEN, Hilden, Germany) and from the tumor tissue using a Puregene Buccal Cell Core Kit B (QIAGEN). For the blood and tumor DNA samples, the entire coding region (exon 1–16) and exon-intron boundaries of the *NF2* gene were amplified by polymerase chain reaction (PCR) using appropriate primer sets. PCR was performed in 25-µl reactions containing 0.2 mM of each dNTP, 10 µM forward and reverse primers, 10× *Taq* buffer, 0.25 U of *Taq* DNA polymerase (Solgent, Daejeon, Korea), and 25 ng of genomic DNA from the blood or tumor, respectively. The PCR conditions consisted of an initial denaturation at 95°C for 15 min, followed by 30 cycles of 95°C for 20 s, 57°C for 40 s, and 72°C for 1 min, with a final 5-min extension at 72°C. The PCR products were confirmed by electrophoresis in 2% agarose gels stained with ethidium bromide (EtBr). For each PCR product, 7 µl were treated with 0.1 U of shrimp alkaline phosphatase (USB, Cleveland, OH, USA) and 1 U of exonuclease I (USB) at 37°C for 1 h, followed by incubation at 80°C for 15 min for enzyme inactivation. The purified PCR products were sequenced in 10-µl reactions containing 5 µM primer, 0.2 µl of ABI Big Dye Terminator v3.1 Cycle Sequencing Kit reagent (Applied Biosystems, Foster City, CA, USA), and 1 µl of 5× buffer. The PCR conditions consisted of 2 min at 95°C, followed by 30 cycles of 95°C for 20 s, 55°C for 20 s, and 60°C for 4 min. The sequencing reaction products were ethanol precipitated, and the pellets were resuspended in 10 µl of HiDi-formamide loading dye. An ABI 3130XL DNA sequencer was used to resolve the products, and the data were analyzed using ABI Sequencing Analysis (v5.0) and SeqScape software (v2.5). The resultant sequence was compared with the *NF2* sequence in GenBank (GenBank ID NM_016418).

### Loss of heterozygosity assay

Loss of heterozygosity (LOH) was determined using flanking and intragenic microsatellite markers in the *NF2* region of chromosome 22. Three microsatellite markers were chosen for the *NF2* gene region from the NCBI database (www.ncbi.nlm.nih.gov): D22S275 is centromeric, D22S929 is intragenic within intron 1 of the *NF2* gene, and D22S268 is telomeric to the *NF2* gene. These three microsatellite markers are tightly linked to the *NF2* gene (mean distance between D22S275 and D22S268 is 0.7 Mb). PCR was performed to genotype the samples, and fluorescently labeled (FAM) markers were used. Each 10-µl reaction contained 0.2 mM of each dNTP, 10 µM labeled forward and reverse primers, 10× *Taq* buffer, 0.25 U *Taq* DNA polymerase (Solgent), and 25 ng of blood genomic DNA or tumor DNA. The PCR conditions consisted of an initial 2 min at 95°C, 35 cycles of 95°C for 20 s, 57°C for 40 s, and 72°C for 30 s, and a final extension at 72°C for 5 min. Subsequently, each PCR product was diluted with distilled water. HiDi-formamide and the GeneScan™ 500 LIZ® size standard (Applied Biosystems) were added to the diluted PCR products, and the samples were loaded (30 cm capillary, POP 7 polymer) in an ABI 3130XL genetic analyzer. The genotypes of the microsatellite markers were analyzed using GeneMapper software (v3.7).

### Methylation-specific PCR (MSP)

Abnormal DNA methylation, *i*.*e*., hypermethylation, in the promoter region, including the CpG island, of the *NF2* gene was examined using bisulfate modification of tumor tissue genomic DNA from all the patients. The bisulfate modification process was performed using an EZ-96 DNA methylation kit (Zymo Research, Orange, CA, USA). Bisulfate converts unmethylated cytosines in DNA into uracil, while methylated cytosines remain unchanged; uracil is then converted into thymidine in the subsequent PCR amplification. To test for methylation, methylation-specific PCR (MSP) was performed using a methylation-specific primer pair (forward 5′-GAGTTATTTTAAAGGAGGCGGGAC-3′ and reverse 5′-GAAACCCCTAAACGACAAC-GAC-3′; 304-bp product), and unmethylation-specific PCR was performed using an unmethylation-specific primer pair (forward 5′-AGTTATTTTAAAGGAGGTGGGATGG-3′ and reverse 5′-AAACAAAACCCCTAAACAACAA-3′; 307-bp product). The primers were designed using the MethPrimer program available at http://www.urogene.org/methprimer/index1.html [10]. Normal genomic DNA was used as the negative control in the MSP, and normal genomic DNA that was methylated using CpG methylase M.Sss1 (New England Biolabs, Beverly, MA) was used as the positive control. The PCR conditions consisted of an initial 5 min at 95°C, 35 cycles of 95°C for 20 s, 57°C for 40 s, and 72°C for 1 min, and a final extension at 72°C for 5 min. The PCR products were confirmed by 2% agarose gel electrophoresis and EtBr staining.

### Statistical analysis

Statistical analysis was performed using the SPSS 18.0 statistics software for Windows (SPSS, Chicago, IL). Student's *t*-test was used to compare the tumor size, clinical growth index, and Ki-67 LI between the patients with and without mutations. *P*-values less than 0.05 were considered significant.

## Results

### Mutational analysis of the *NF2* gene

Mutational analysis of the entire coding region, *i*.*e*., exons 1 to 16, and the flanking intronic sequences of the *NF2* gene was performed using direct sequencing using genomic DNA from the blood and tumors of 30 patients with VS. No germ-line mutations were found in blood DNA, whereas 18 mutations were found in tissue DNA. These mutations included frameshift, nonsense, in-frame, missense, and splice site mutations. Nine different mutations (five single-nucleotide deletions or insertions, two multiple-nucleotide deletions in the coding region, and two several nucleotide deletions in the coding region and flanking intron sequences) were identified. Six of the eight frameshift mutations resulted in the premature termination of translation and the other two frameshift mutations generated a truncated protein via exon skipping caused by the loss of a splice acceptor or donor site. One 78 bp deletion mutation led to an in-frame deletion. In addition, seven nonsense mutations were identified that caused the generation of a prematurely terminated protein due to an early stop codon. Among the patients, two (cases 4 and 24) had two different mutations each. One patient had a missense mutation of a thymine to guanine transition at nucleotide 136 in exon 2, causing an amino acid change from leucine to arginine. One patient had a splice site mutation at a donor site, and the splice site mutation led to a substitution of guanine for adenine (GT to AT) at the donor site in intron 3, thus causing an in-frame mutation by skipping exon 3. Consequently, all of the mutations in patients with VS were **somatic** mutations of the *NF2* gene.

### Loss of heterozygosity

**Figure 1:**
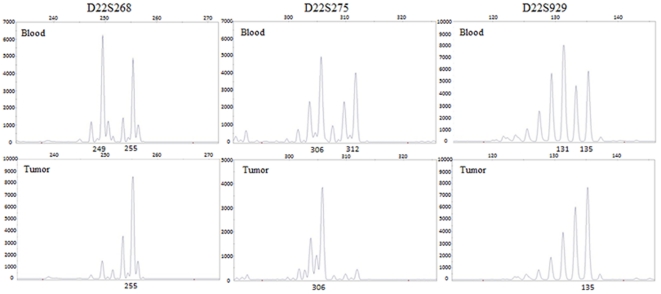
Loss of heterozygosity analysis of case No.1. A comparison of lymphocyte DNA and tumor DNA using the flanking, D22S268 and D22S275, and intragenic, D22S929, microsatellite markers in the *NF2* gene region. In case, three markers showed loss of heterozygosity by allele loss in the tumor DNA.

Genomic DNA (blood and tumor DNA) from all 30 patients was investigated for LOH using three highly polymorphic microsatellite markers near the *NF2* gene region: two flanking markers (D22S268 and D22S275) and one intragenic marker within intron 1 (D22S929). Comparative analysis of the LOH of genomic DNA from blood and tumor samples showed heterozygous blood DNA and homozygous tumor DNA (Fig. 1). Of the 30 patients with VS, 9 (30%) had LOH, and 8 of the 9 showed LOH with the flanking marker D22S268 and the intragenic marker D22S929. Every patient with LOH had the D22S275 flanking marker. Thus, 21 of the 30 patients (70%) did not show LOH in their tumor DNA (Table 1).

**Table 1:** Loss of heterozygosity (LOH) and mutation in sporadic vestibular schwannomas.

No	Exon	DNA sequence alteration	Codon change	Consequence	LOH		
					D22S268	D22S275	D22S929
1	2	c.136T>G (p.L46R)	46	Missense	+	+	+
2	3	c.363+1G>A	-	Splicing donor site	+	+	+
3	-	-	-	-	+	+	+
4	2, 3	c.232_233 ins G c.303T>A (p.Y101X)	78 101	Frameshift, Nonsense			
6	3	c.280 del T	94	Frameshift	+	+	+
8	2	c.122G>A (p.W41X)	41	Nonsense			
10	13	c.1379 del T	460	Frameshift			
12	2	c.169C>T (p.R57X)	57	Nonsense	+	+	+
13	12	c.133 C>G (p.S444X)	444	Nonsense	+	+	+
14	-	-	-	-	+	+	+
15	2	c.154_175del22	51–59	Frameshift			
17	10	c.955C>T (p.Q319X)	319	Nonsense			
21	10	c.963_964 ins A	322	Frameshift		+	
22	14	c.1568_1574+7del14	523–525+intron7bp	Frameshift			
24	7, 8	c.616G>T (p.E206X) c.694_771del78	206 232–257	Nonsense In-frame deletion			
27	4	c.364-8_408del53	Intron8bp+122–136	Frameshift			
29	2	c.169C>T (p.R57X)	57	Nonsense			
30	4	c.394 delT	132	Frameshift	+	+	+

### CpG island hypermethylation of the *NF2* gene

Methylation and unmethylation primers were designed for the region approximately 350 bp to 650 bp upstream of the start codon, which includes the CpG island, using the program MethPrimer (Li and Dahiya, 2002). The methylation state of the promoter region was examined using MSP and unmethylation-specific PCR (USP). The promoter region of the bisulfite-treated DNA of all patients and the negative control (NC) was amplified using USP, and the PCR products were identified using electrophoresis. The bisulfite-treated DNA positive control (PC) was amplified using only MSP. The results demonstrated that the expression of the *NF2* gene in all of the VS patients was not repressed by hypermethylation of the promoter region.

### Genetic alterations and tumor behavior

The average tumor sizes in the patients with mutations or LOH and patients without mutations and LOH were 16.11 and 20.83 cm, respectively. The average clinical growth index (CGI) values were 13.22 and 12.92, and the average Ki-67 labeling index (LI) values were 2.21 and 2.54, respectively. There was no significant difference in tumor size (*p* = 0.105), CGI (*p* = 0.878), or Ki-67 LI (*p* = 0.372) between the patients with mutations or LOH and the patients without mutations and LOH. Thus, there was no correlation between the nature of the *NF2* gene mutation, including LOH, and tumor behavior.

## Discussion

This study investigated the genetic alterations in sporadic VS, including mutations, loss of heterozygosity (LOH), and epigenetic alterations of the *NF2* gene, and compared these alterations with the tumor behavior. The *NF2* gene is frequently mutated in NF2-related vestibular schwannomas [1], [2], and mutations in this gene have also been found in sporadic unilateral schwannomas [8], [11], [12]. The rate of mutations is higher in unilateral schwannomas than in familial schwannomas. We found 18 genetic alterations (60%) in 30 Korean patients with sporadic VS, which was in agreement with the 53–70% detection rate reported in previous studies [9].

The majority of the mutations (88%) in our study were protein-truncating mutations such as frameshift or nonsense mutations. Among constitutional NF2 mutations, nonsense mutations are significantly more common than frameshift mutations in NF2 patients, whereas frameshift mutations are significantly more common than nonsense mutations among somatic NF2 mutations in sporadic vestibular schwannomas [13]. We also found that frameshift mutations (53%) were more common than nonsense mutations (35%) [14] . Donor splice site mutations and missense mutations were rare, which was consistent with other studies [7], [8]. According to Knudson's two-hit hypothesis [15], functional inactivation of a specific tumor-suppressor gene requires two separate genetic hits in the same gene in the same cell. For a sporadic tumor, the probability of both mutations occurring in the same cell is extremely small [16]. Two patients (cases 4 and 24) each had two different mutations that inactivated the *NF2* gene. It is possible that LOH represents the second hit on the remaining allele. Nine patients (30%) in our study had chromosome 22 LOH, and seven patients had both LOH and mutations. In our study, nine patients (30%) had two hits, and another nine patients (30%) had only one hit (mutation or LOH). It has been shown that mitotic recombination plays a role in the occurrence of LOH, even though the rate of mitotic recombination is relatively low in sporadic VS compared to NF2-related schwannomas [17]. In the other cases, we postulate that other mechanisms, such as different genetic abnormalities or epigenetic factors, were involved in the pathogenesis of the vestibular schwannomas.

To search for other genes associated with vestibular schwannoma tumorigenesis, Welling et al. [18] used cDNA microarray analysis to evaluate gene expression profiles. They identified a number of dysregulated genes involved in cell signaling, cell division, and angiogenesis. Caye-Thomasen et al. [19] examined the gene expression profile in vestibular schwannomas using a microarray chip and identified 78 dysregulated genes involved in the cell cycle, morphogenesis, development, adhesion, differentiation, death, extracellular matrix, and protein binding.

Methylation of the promoter-associated CpG island is a possible mechanism of tumor-suppressor gene inactivation in a variety of human tumors [4]. Lomas *et al.* [20] proposed that aberrant NF2 hypermethylation may participate in the development of a significant proportion of sporadic meningiomas. However, Kullar *et al.* [21] recently reported that hypermethylation of the CpG island of the *NF2* gene was rare in sporadic vestibular schwannomas. None of our patients showed repressed expression of the *NF2* gene due to hypermethylation of the promoter region. Instead, methylation of other tumor-related genes may have played a role in the development of these vestibular schwannomas [22].

Several studies have examined whether genotype predicts the disease severity [23]. Irving et al. [7] found no correlation between the nature of the *NF2* gene mutation and tumor behavior. Conversely, Bian *et al*. [6] reported a significant relationship between tumor behavior and genetic alterations in Chinese patients. Consequently, we investigated whether there was a racial difference in the genotype-phenotype correlation in sporadic vestibular schwannomas. To quantify tumor behavior, the previously mentioned studies used the clinical growth index (CGI). However, the clinical growth index is far from ideal because of the variability of the clinical symptoms and the uncertainty of their duration. Therefore, we used tumor size and CGI as a measure of tumor behavior. In the present study, we found no difference in tumor behavior between Korean patients with and without *NF2* genetic alterations, suggesting that there is no racial difference.

In summary, the present study found 16 cases of mutations and 9 cases of LOH in the *NF2* genes of 30 Korean patients with sporadic VS. However, no aberrant hypermethylation of the *NF2* gene was detected, and no clear genotype/phenotype correlation was identified in our study. Therefore, other factors are likely to contribute to tumor formation and growth.
